# Organizational Facilitation of Latino Substance Use Disorder Treatment: Impact of COVID-19

**DOI:** 10.1089/heq.2022.0184

**Published:** 2023-11-20

**Authors:** Ruth Campbell, Smita Dewan

**Affiliations:** ^1^ Connections Counseling PLLC, New Paltz, New York, USA.; ^2^ Department of Human Services, NYC College of Technology, City University of New York, Brooklyn, New York, USA.

**Keywords:** opioid epidemic, health equity, Latino/Hispanic, participatory research, substance use disorder, telehealth

## Abstract

**Purpose:**

Continued high opioid overdose death rates in the United States and increasing New York State (NYS) Latino opioid overdoses make the facilitation of Latino access to NYS substance use disorder (SUD) treatment essential. SUD treatment facilities in NYS sustained an estimated 37% decrease in Latino enrollment during phase one of the pandemic. This study invited NYS SUD service providers to describe ways in which SUD organizations facilitated Latino SUD treatment prior to and during phase one of the pandemic.

**Methods:**

Using an individual and community interaction framework of vulnerability and a description of organizational enabling resources in four domains, this study used a cross-sectional descriptive design to investigate the levels of organizational facilitators for Latino SUD treatment access and the impact of the pandemic on these organizational facilitators. A convenience sample of 470 NYS SUD clinicians participated in the study.

**Results:**

The outcomes suggest an overall erosion of organizational enabling resources during the pandemic. Erosion was greatest in areas with a higher Latino population density in the domains of insured/immigration/legal information and culture. A pattern of strengthening resources in areas with lower Latino population density in the domains of language and telehealth access has defied the overall pattern of deterioration. The increase in telehealth did not cross the digital divide to stop the decrease in Latino enrollment and did not compensate for the overall erosion of access facilitators.

**Conclusions:**

The overall outcomes suggest opportunities to explore local variations in resource health. Recommendations to improve health equity include the use of participatory research to assess community needs and the implementation of community partnerships to address systemic barriers and rebuild equitable addiction services.

## Introduction

Vulnerable communities face difficulties in accessing care during infectious disease epidemics as well as devastating socioeconomic problems. This combination leads to poor health outcomes^1,2^ for communities such as the Latino community in New York State (NYS) during the convergence of COVID-19 and the opioid epidemic. Fostering the well-being of the impoverished, refugees, minorities, and the uninsured during these crises has presented a major challenge to the state. The NYS Latino community has been particularly hard hit by the opioid epidemic,^3,4^ despite efforts on the part of the state to prevent overdose deaths.^5–7^

Nationally, overdose deaths for Latinos have nearly tripled since 2010; much of the increase is ascribed to the availability of synthetic opioids like fentanyl.^8^ Recent NYS data list the opioid overdose death rate at 24.5 per 100,000 people, 14% higher than the previous year.^9^

Many barriers to Latino help-seeking for substance use issues such as opiate use disorders are systemic.^10–14^ This study sought to investigate NYS organizational efforts to facilitate care for Latino community members seeking help for substance use disorders (SUDs). It also explored the effect of the COVID-19 pandemic on these efforts. Lessons learned from the NYS experience in addressing systemic barriers to Latino help-seeking may be instructive since New York is in the top 10 U.S. states with the highest Latino population density.^15^

### Conceptual framework

**FIG. 1.**
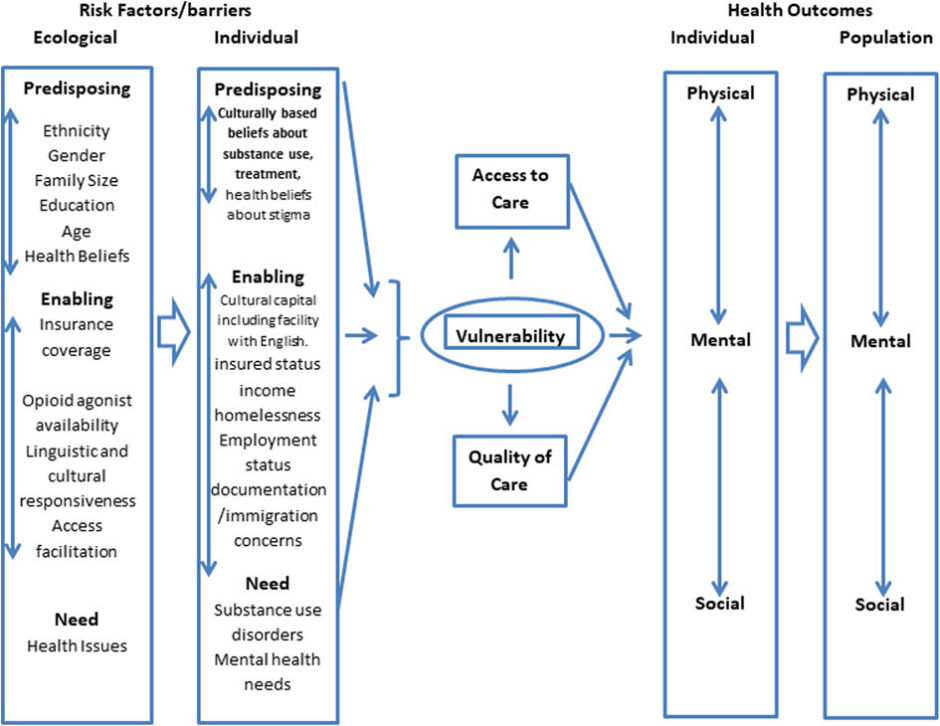
Vulnerability framework.

Based on a systemic understanding of the factors influencing health, a vulnerability framework,^1^ models interacting environmental and individual factors that cumulatively result in individual susceptibility to poor health outcomes (Fig. 1). The model has been tailored to detail predisposing, enabling, and need factors for the Latino community.

Culturally based beliefs about substance use,^16–18^ about treatment,^11,19,20^ and health beliefs regarding the stigma of substance use problems^16,18,21^ are predisposing factors, indicating the propensity of individuals to use services.

Enabling factors, indicating resources available for the use of services, may include cultural capital, including facility in English,^20,22–24^ homelessness,^25^ employment status,^16,25^ poverty,^22^ insured status,^10,12,16,18^ and documentation/immigration issues.^11,18,21,22^ Organizational enabling factors may include insurance coverage for services,^22,24,26^ access facilitation,^22^ availability of opioid agonist therapy,^25^ and linguistic and cultural responsiveness.^14,16–18,21,22,27^ Need factors for this population include help with SUDs and mental health problems that may co-occur with SUDs.^23,28^

During phase 1 of the pandemic, NYS addiction service organizations, specialty facilities for state SUD treatment,^29^ faced two convergent crises that stressed service provision. Understanding that organizational facilitators of Latino help-seeking could be crucially important to health outcomes, the nonprofit organization initiating this study asked counselors employed by NYS addiction service organizations to respond to questionnaires concerning organizational facilitators of Latino participation in addiction treatment.

### Problem statement and hypotheses

Hypotheses concerning the levels of organizational facilitators for Latino SUD treatment in NYS addiction service organizations and how levels were affected by the pandemic are as follows:

1. Levels of the following facilitators decrease with the onset of the pandemic compared to the prepandemic era: (a) language, (b) legal/financial/insured status, (c) culture, and (d) access to services.
2. Changes in the level of the following facilitators differ according to the categories of Latino population density: (a) language, (b) legal/financial/insured status, (c) culture, and (d) access to services.
3. There is a positive correlation between the Spanish language competence of the practitioner and the caseload percentage of Latino clients during the prepandemic and pandemic periods.

## Methods

### Participants

Study investigators used a sampling frame list of e-mail addresses of active clinicians, certified by the Office of Addiction Services and Supports (NYSOASAS), to develop a convenience sample of participants.

All NYS clinicians older than 21 years of age who held Certified Alcohol and Substance Abuse Counselor (CASAC) certificates were e-mailed invitations to participate in a free six-credit (CEU) training opportunity in English and Spanish, which was part of a research study on how addiction treatment was facilitated. Completion of the study questionnaires was a requirement for the training.

Special notices were placed on the NYSOASAS website, and phone contact with each of the directors of the NYSOASAS facilities was attempted to alert clinicians and clinic directors to the opportunity. Those who registered signed the informed consent, indicating that they understood that they were participating in the research study. Over 6,000 clinicians held the CASAC at the time of initiation of the study planning; however, it was noted that the number of clinicians with CASAC credentials changed daily (personal communication; M. McKeown, July 3, 2018). Copies of the survey instrument can be obtained upon request from the corresponding author.

### IRB approval

Ethical protection of research subjects and approval of research protocols were carried out by the Western Copernicus Group Independent Research Board.

### Data collection

Data were collected consistently throughout the 2018–2022 years; the free six-CEU course was continually offered to NYS clinicians. When data were harvested in 2022, the prepandemic (2018–2019) and pandemic (March 2020–2022) data were separated for analysis. Data collection was performed using questionnaires administered electronically via SurveyMonkey.

The method resulted in the collection of 470 questionnaires completed by NYS addiction services clinicians, which documented data on the variables of interest in the study. Of the 470 completed questionnaires, 305 responded to the organizational resource questions. Consequently, this study included findings from completed surveys (with organizational facilitator comments) of 305 clinicians.

### Measures

The study collected data on the following independent variables: Time Period, Location Population Density, and Spanish Language Competence. Data were also collected on dependent variables: Organizational Facilitators of Latino involvement in SUD treatment, and Caseload Percentage of Latinos in treatment. Clinicians' sociodemographic information was collected for descriptive purposes.

#### Independent variables

##### Time period

Two categories of time period were compared: 2018–2019 data were placed in the prepandemic category, and March 2020–April 2022 data were placed in the phase 1 pandemic category.

##### Location population density

Clinician responses were categorized into two groups, upstate (county Latino population density <10%) and downstate (county Latino population density >10%), based on the Latino population density in the county of the clinician's practice.^30^

##### Spanish language competence

To determine whether there was an organizational effort to culturally “match” clinicians to Latino clients, a cultural adaptation strategy adopted by some addiction facilities,^17^ we adapted a scale of acculturation that used Spanish language as the only measured parameter, the Short Acculturation Scale for Hispanics (SASH).^31^ We used the scale as a measure of the ability of clinicians to speak Spanish and potentially to understand the culture of Latinos seeking treatment. The advantages of the scale are as follows: it was developed with populations of Spanish speakers that are similar to Spanish-speaking populations in NYS, it is entirely based on the context of Spanish language use, and it has a Cronbach's alpha of 0.92, indicating internal consistency.^32^ Higher scores are inversely associated with individual ability to speak Spanish. This particular use (for the measurement of Spanish language competence and a first approximation to cultural competence) has no precedent in the literature.

#### Dependent variables

##### Organizational facilitators

Four domains capture the representation of this variable: language, legal/immigration/insurance status, cultural, and geographical/access. Each domain was represented by four closed-ended questions and one open-ended question. Clinicians were invited to remark on the resources that might facilitate Latino utilization of treatment with a final open-ended question. For example, a representative question is: “LANGUAGE: The resources listed below might help Latinos overcome language barriers in order to get help. Please check the ones that your organization offers. If you know of other ways that your organization helps Latinos overcome language barriers, please list them in the space marked ‘comments.’”

##### Caseload percentage

This variable was represented by the percentage of Latinos in each SUD counselor caseload, as estimated by the counselor.

Other sociodemographic and practice measures were formulated as suggested by the research standards.

### Analysis

A cross-sectional descriptive design allowed the comparison of the levels of organizational facilitators of SUD treatment for Latinos in phase 1 of the pandemic to prepandemic levels.

A bivariate analysis was conducted to test all research hypotheses. Depending on the level of measurement of the variables, t-tests, chi-square analyses, and Pearson's correlations were used to compare the pandemic with prepandemic values.

Chi-square analyses compared organizational facilitator data during the pandemic and prepandemic periods. The outcome of this analysis tested Hypotheses 1a–1d, concerning the change in the level of organizational facilitators during the pandemic. Chi-square analyses compared downstate and upstate resource levels in the two time periods to test the hypothesized differential in the change of facilitators in the two categories of Latino population density during the pandemic compared to the prepandemic period (Hypothesis 2a–d).

A Pearson correlation of Spanish language competence and caseload percentage of Latino clients tested the hypothesized correlation between clinician Spanish language competence and the percentage of Latino clients in clinician caseloads in the two periods (Hypothesis 3).

## Results

### Sample demographics and practice characteristics

Sampled clinicians in direct practice were female (70.4%), Caucasian (57.6%), had an average age of 51.6 years (SD=11.9), had an education level of master's or above (51.6%), and were certified to practice SUD treatment with CASAC certification (92.8%). Clinicians had an average of 15.4 (SD=9.4) years in practice, and were generally English-only speaking (*M*=19.1; SD=2.2). Clinicians noted that they generally worked in an outpatient setting (41.8%), offered SUD treatment (75.2%), and, of those who practiced, had an average of 22.6% Latino clients on their caseload.

#### Prepandemic/pandemic demographic comparison

There was a significant reduction in black employment in clinical treatment roles, from 17.9% in the prepandemic period to 12.4% in phase 1 of the pandemic [χ^2^(3,84)=7.94, *p*=0.047]. There was also a significant increase in the percentage of counselors with certification to work with SUDs, from 91.2% in the prepandemic period to 99.0% during phase 1 of the pandemic [χ^2^(1,83)=5.69, *p*=0.017]. There were no significant changes in age, gender, Spanish language competence, or education level of the surveyed clinicians during the two periods.

##### Correlation of Spanish language competence and caseload percent of Latinos

Spanish language competence, as measured by the SASH scale, was significantly and positively (inverse measure) correlated with the clinician caseload percentage of Latino clients, both before the pandemic (*r*=−0.50; *p*<0.001) and during the pandemic phase 1 (*r*=−0.52; *p*<0.001), indicating that those clinicians who had Spanish language competence had higher caseload percentages of Latino clients in both time periods.

#### Prepandemic/pandemic comparison of numbers of Latinos in treatment

There was a significant overall reduction (36.9%) in the caseload percentage of Latinos in treatment from 23.8% in the prepandemic period to 15.0% during phase 1 of the pandemic [*t*(264)=−2.10, *p*=0.018]. This reduction was reflected in reports by upstate and downstate clinicians.

### The prepandemic/pandemic facilitation of SUD care

Results of chi-square analyses that assessed the relationship between time periods, and organizational resources are shown in Tables 1–4.

**Table 1.** Language Resources: Comparison of Prepandemic/Pandemic Clinic Resources Reported by Surveyed Clinicians (*N*=456) with Additional Significant Upstate and/or Downstate Data

	All NYS CASACs		Prepandemic		Pandemic		χ^2^
	***N***	%	***n***	%	***n***	%	
Language, total	456		371		85		
Translators	124	27.2	90	24.3	34	40.0	8.66^**^
Clinicians who speak Spanish	154	33.8	112	69.8	42	49.4	11.43^**^
Signage in Spanish	88	19.3	60	16.2	28	32.9	12.49^***^
None of the above	74	16.2	42	11.3	32	37.6	35.26^***^
Upstate			138		36		
Translators			48	34.8	21	58.3	6.62^**^
Clinicians who speak Spanish			30	21.7	17	47.2	9.40^**^
Signage in Spanish			24	17.4	16	44.4	11.80^***^
None of the above			19	13.8	11	30.6	5.64^*^
Downstate			224		48		
Translators			40	17.9	13	27.1	2.15
Clinicians who speak Spanish			80	35.7	24	50.0	3.42^*^
Signage in Spanish			35	15.6	12	25.0	2.43
None of the above			23	10.3	21	43.8	32.68^***^

^*^ *p*<0.05, ^**^p<0.01, ^***^p<0.001.

CASAC, Certified Alcohol and Substance Abuse Counselor; NYS, New York State.

**Table 2.** Immigration/Insured/Legal Status Resources: Comparison of Prepandemic/Pandemic Clinic Resources Reported by Surveyed Clinicians (*N*=456) with Additional Significant Upstate and/or Downstate Data

	All NYS CASACs		Prepandemic		Pandemic		χ^2^
	***N***	%	***n***	%	***n***	%	
Total status resources	456		371		85		
Legal help phone nos	99	21.7	76	20.5	23	27.1	1.76
Info re undocumented rights	71	15.6	54	14.6	17	20.0	1.56
Info re svc coverage	101	22.1	77	20.8	24	28.2	2.24
None of the above	104	22.8	60	16.2	44	51.8	49.76^***^
Upstate			138		36		
Legal help phone nos			27	19.6	12	33.3	3.11
Info re undocumented rights			18	13.0	8	22.2	1.89
Info re svc coverage			25	18.1	13	36.1	5.42^*^
None of the above			30	21.7	14	38.9	4.45^*^
Downstate			224		48		
Legal help phone nos			48	21.4	11	22.9	0.05
Info re undocumented rights			35	15.6	9	18.8	0.29
Info re svc coverage			51	22.8	11	22.9	0.00
None of the above			30	13.4	29	60.4	51.46^***^

^*^ *p*<0.05, ^***^p<0.001.

**Table 3.** Cultural Resources: Comparison of Prepandemic/Pandemic Clinic Resources Reported by Surveyed Clinicians (*N*=456) with Additional Significant Upstate and/or Downstate Data

	All NYS CASACs		Prepandemic		Pandemic		χ^2^
	***N***	%	***n***	%	***n***	%	
Cultural	456		371		85		
Neighborhood resource phone nos	118	25.9	85	22.9	33	38.8	9.13^**^
Clinic outreach	57	12.5	45	12.1	12	14.1	0.25
Childcare in Spanish	39	8.6	32	8.6	7	8.2	0.01
None of the above	117	25.7	73	19.7	44	51.8	37.30^**^
Upstate			138		36		
Neighborhood phone nos			35	25.4	17	47.2	6.51^**^
Clinic outreach			13	9.4	5	13.9	0.62
Childcare in Spanish			12	8.7	4	11.1	0.43
None of the above			32	23.3	16	44.4	6.46^**^
Downstate			224		48		
Neighborhood phone nos			48	21.4	16	33.3	3.11
Clinic outreach			31	13.8	7	14.6	0.02
Childcare in Spanish			19	8.5	3	6.3	0.27
None of the above			41	18.3	27	56.3	30.36^***^

^**^ *p*<0.01, ^***^p<0.001.

**Table 4.** Access Resources: Comparison of Prepandemic/Pandemic Clinic Resources Reported by Surveyed Clinicians (*N*=456) with Additional Significant Upstate and/or Downstate Data

	All NYS CASACs		Prepandemic		Pandemic		χ^2^
	***N***	%	***n***	%	***n***	%	
Access, total	456		371		85		
Telehealth	59	12.9	33	8.9	26	30.6	28.89^***^
Late clinic hours	103	22.6	82	22.1	21	24.7	0.27
Transportation	87	19.1	63	17.0	24	28.2	5.67^*^
None of the above	93	20.4	60	16.2	33	38.8	21.86^***^
Upstate			139		36		
Telehealth			14	10.1	12	33.3	12.08^***^
Late clinic hours			30	21.7	8	22.2	0.00
Transportation			29	21.0	14	38.9	4.90^*^
None of the above			24	17.4	12	33.3	4.42^*^
Downstate			224		48		
Telehealth			18	8.0	13	27.1	14.20^***^
Late clinic hours			51	22.8	13	27.1	0.41
Transportation			33	14.7	10	20.8	1.11
None of the above			35	15.6	21	43.8	19.13^***^

^*^ *p*<0.05, ^***^p<0.001.

#### Comparison of facilitation of SUD care by language factors

A comparison of prepandemic and pandemic language resources is presented in Table 1. Before the pandemic, ∼89% of the surveyed NYS clinicians reported that their clinics had language resources to facilitate Latino client involvement in treatment. Clinics and clinicians had developed creative methods to overcome these language barriers.

Upstate clinicians, more often English-only speaking, most commonly (34.8%) mentioned translators; downstate clinicians, most commonly (35.7%) mentioned Spanish-speaking clinicians who met the needs of Spanish-speaking clients. Providers with sufficient numbers of Spanish-speaking clinicians matched the Spanish-speaking clientele with the clinicians. Providers with more Spanish-speaking clients than their clinics could easily serve used strategies that increased the client-to-provider ratio; they referred Latinos who could not speak English well to “resources that can assist them in their language,” ran groups in Spanish, used adjunct staff, or used technical assistance, such as phone translation, CRYACOM, translator lines, or automatic translators. Referrals were the second most commonly mentioned resource that provided language facilitation for Spanish speakers.

The percentage of clinicians who reported the availability of language services decreased significantly from 89% during the prepandemic period to 62% during phase 1 of the pandemic period [χ^2^(1,85)=35.26, *p*=0.000]. Downstate [χ^2^(1,48)=32.68, *p*=0.000] and upstate [χ^2^(1,36)=5.64, *p*=0.018] clinicians reported a significant decrease in language services. The clinical staff continued to mention the use of referrals to Spanish-speaking programs during the pandemic period in open-ended comments. Technological services such as language lines or 711 Relay services helped English-speaking clinicians cross the language barrier to assist Spanish-speaking clients. Groups and adjunct staff continued to be used to good advantage to allow Spanish speakers to access treatment.

#### Comparison of facilitation of SUD care by legal/immigration/insured status factors

A comparison of organizational facilitators for legal, immigration, and insured status barriers during the two time periods is presented in Table 2. Before the pandemic, over 80% of the surveyed NYS clinicians declared that their clinics facilitated Latino client involvement in treatment by providing legal, immigration, and insurance information. This included information on legal help, undocumented rights, and insurance coverage. The pandemic period ushered in a dramatic decrease in these services [χ^2^(1,85)=49.76, *p*=0.000]. Before the pandemic, 84% of clinicians reported that their clinics had these types of services compared to only 48% during the pandemic. This study found that organizational erosion in the immigration/insured/legal status facilitators (42.5%) was steeper than that in any other domain during the convergence of crises.

#### Comparison of facilitation of SUD care by cultural factors

Before the pandemic, the majority (80.3%) of surveyed clinicians reported that their clinics facilitated Latino involvement by means of cultural resources. Upstate (25.4%) and downstate (21.4%) clinicians cited a list of phone numbers for welcoming Latinos to local neighborhoods as the most commonly provided clinic cultural resource. Clinic outreach to the community and childcare in Spanish were common organizational facilitators. Downstate clinics often provided outreach to the churches or festivals that Latinos might attend.

During phase 1 of the pandemic, upstate (44%) and downstate (56%) clinicians reported significantly diminished cultural resources [χ^2^(1,85)=37.33, *p*=0.000]. A comparison of the cultural resources during the two periods is presented in Table 3.

#### Comparison of facilitation of SUD care by geographical/access factors

A comparison of organizational resources that facilitated access to SUD treatment during the two time periods is presented in Table 4. Before the pandemic, over 80% of upstate and downstate clinicians declared that their clinics had resources that could facilitate Latino access and involvement.

During phase 1 of the pandemic, this picture shifted radically as clinics, clients, and clinicians had to close down, lock down, quarantine and work from home. A significant number [χ^2^(1,85)=21.86, *p*=0.000] of clinicians reported that their clinics stopped offering these services.

In clinics that maintained services, telehealth services burgeoned. Upstate [χ^2^(1,36)=12.08, *p*=0.001] and downstate clinicians [χ^2^(1,48)=14.20, *p*=0.000] reported a significantly increased provision of telehealth, replacing physical access with virtual access.

## Discussion

Before COVID-19, health delivery cultural competence^33^ was demonstrated by NYS treatment organizations. Organizational enabling resources included accommodation of Spanish language needs, cultural facilitators, provision of information about neighborhood, immigration, legal, and insurance status resources, and access facilitation for Latino clinic attendees.

### Study limitations

The outcomes of this study have limited generalizability due to the convenience sampling method. Study outcomes may point to trends but cannot be regarded as representative at the state or local level.

### Erosion and strengthening of organizational facilitators during COVID-19

The study outcomes suggest that NYS SUD organizational enabling resources eroded during the pandemic (Hypothesis 1), and deterioration was worse in areas with higher (>10%) Latino population density (Hypothesis 2). Organizational matching of Spanish-speaking clients to clinicians with Spanish language competence continued undiminished through the two time periods (Hypothesis 3). As shown in Tables 1–4, resources in downstate counties with an average Hispanic population density of over 10% deteriorated the most, and the deterioration was worst in the domains of legal/immigration/insurance status and culture.

However, this pattern was more complicated than that suggested by the overall analysis. As seen in Tables 1–4, data demonstrates resource increase in subdomains in upstate and downstate areas during the pandemic, while services decreased overall. Resource strengthening was greatest in areas with lower Latino population density in the domains of language (signage in Spanish) and telehealth. This paradoxical finding suggests that some clinics increased their resources during the pandemic, while most clinics stopped offering services in the listed domains.

### Implications for health equity

The outcomes of this study suggest that the disparity that exists in NYS health care delivery deepened during the pandemic, and that health care access varied widely at the community level. The 37% overall decrease in the number of Latinos receiving SUD treatment during the pandemic suggests that barriers to treatment were too steep for Latinos to obtain assistance through traditional SUD treatment avenues. A comment by one downstate clinician about Hispanics being “at risk for incarceration” highlights only one potential outcome of this organizational failure.

Treatment access was significantly affected by the rise in telehealth, but the increase was not sufficient to counter the overall drop in access resources. Evidence from the steep decline in Latino enrollment suggests that telehealth could not reach the Latino community across the digital divide. These findings offer opportunities for further research.

Of the many strategies used to address health disparities,^1,2,34^ study outcomes concerning local service variation underscore the potential efficacy of engaging the community in finding solutions to community problems. The community partnership tradition has often been a bellwether for policy development at state and federal government levels. For NYS addiction services, implementing participatory research and community partnerships could begin the preparation to craft appropriate community-focused solutions to statewide problems, such as continuing high overdose death rates. By focusing on enabling facilitators, a resilient organizational response can develop an awareness of disparity, establish service relevance, and re-establish equitable addiction services.

## Abbreviations Used

CASAC: Certified Alcohol and Substance Abuse Counselor
NYS: New York State
SASH: Short Acculturation Scale for Hispanics
SUD: substance use disorder
